# Spatiotemporal Changes in Pulmonary Tuberculosis Incidence in a Low-Epidemic Area of China in 2005-2020: Retrospective Spatiotemporal Analysis

**DOI:** 10.2196/42425

**Published:** 2023-03-08

**Authors:** Qi Zhang, Huan Ding, Song Gao, Shipeng Zhang, Shiya Shen, Xiaoyan Chen, Zhuping Xu

**Affiliations:** 1 Department of Chronic Communicable Disease The Affiliated Wuxi Center for Disease Control and Prevention of Nanjing Medical University, Wuxi Center for Disease Control and Prevention Wuxi China

**Keywords:** pulmonary tuberculosis, spatial analysis, temporal analysis, epidemiology, China

## Abstract

**Background:**

In China, tuberculosis (TB) is still a major public health problem, and the incidence of TB has significant spatial heterogeneity.

**Objective:**

This study aimed to investigate the temporal trends and spatial patterns of pulmonary tuberculosis (PTB) in a low-epidemic area of eastern China, Wuxi city, from 2005 to 2020.

**Methods:**

The data of PTB cases from 2005 to 2020 were obtained from the Tuberculosis Information Management System. The joinpoint regression model was used to identify the changes in the secular temporal trend. Kernel density analysis and hot spot analysis were used to explore the spatial distribution characteristics and clusters of the PTB incidence rate.

**Results:**

A total of 37,592 cases were registered during 2005-2020, with an average annual incidence rate of 34.6 per 100,000 population. The population older than 60 years had the highest incidence rate of 59.0 per 100,000 population. In the study period, the incidence rate decreased from 50.4 to 23.9 per 100,000 population, with an average annual percent change of –4.9% (95% CI –6.8% to –2.9%). The incidence rate of pathogen-positive patients increased during 2017-2020, with an annual percent change of 13.4% (95% CI 4.3%-23.2%). The TB cases were mainly concentrated in the city center, and the incidence of hot spots areas gradually changed from rural areas to urban areas during the study period.

**Conclusions:**

The PTB incidence rate in Wuxi city has been declining rapidly with the effective implementation of strategies and projects. The populated urban centers will become key areas of TB prevention and control, especially in the older population.

## Introduction

Tuberculosis (TB) is a chronic communicable disease caused by the bacillus *Mycobacterium tuberculosis*, which spreads across the population via the respiratory tract [1,2]. The pathogen most typically invades the lungs (pulmonary tuberculosis [PTB]), though it can affect any organs of the body (extrapulmonary TB) [3,4]. With high infection rates, morbidity, mortality, drug resistance, and HIV coinfection, TB still remains one of the most intractable public health issues, particularly in low- and middle-income countries [5]. According to the *Global Tuberculosis Report 2020* by the World Health Organization [1], there were approximately 10.0 million (range 8.9-11.0 million) people with TB worldwide, of whom 54.6% (n=5.5 million) were from 5 countries, namely India (26.0%), Indonesia (8.5%), China (8.4%), Philippines (6.0%), and Pakistan (5.7%).

With the second-largest TB burden, the Chinese government has taken effective national prevention and control strategies to combat TB. Since 1991, a pilot TB control strategy, the directly observed treatment and short-course (DOTS) strategy, was implemented in several provinces, and the government expanded the DOTS strategy nationwide by 2005 [6]. Moreover, the government revised the Law of the People’s Republic of China on the Prevention and Treatment of Infectious Diseases, and the Chinese Center for Disease Control and Prevention developed the nationwide Notifiable Infectious Disease Reporting Information System, which is the largest internet-based disease reporting system after the severe acute respiratory syndrome outbreak [7]. With the implementation of various TB prevention and control measures, the morbidity and mortality of TB in China have decreased dramatically [8,9]. The incidence of PTB and smear-positive TB in China dropped from 611 and 170 per 100,000 population in 1990 to 442 and 59 per 100,000 population in 2010, respectively [6]. However, due to differences in the population, climate, and socioeconomic factors, the prevalence of PTB in China has significant spatial heterogeneity [8,10,11]. According to the fifth national TB epidemiological survey in 2010, the prevalence of PTB in the eastern, central, and western regions of China was 291, 463, and 695 per 100,000 population, respectively [12]. In addition, the prevalence was significantly higher in rural areas (569 per 100,000 population) than in urban areas (307 per 100,000 population). Another study [13] based on the reported PTB cases in China showed that the reported incidence of PTB in 2019 was 55.6 per 100,000 population in China, and the incidence in Jiangsu Province was 31.3 per 100,000 population, which was one of the lowest incidences nationwide and much lower than that in the Tibet Autonomous Region (182.4 per 100,000 population). In previous studies, researchers have analyzed the spatial epidemiological characteristics of PTB, which are of great significance for the effective allocation of limited health resources [14-17]. However, previous studies [15-17] mainly focused on areas with high PTB incidence in China; however, they seriously ignored the evolution of epidemiological characteristics of PTB in low-epidemic areas. Nie et al [18] analyzed the spatial distribution characteristics of PTB cases in Hefei City from 2009 to 2020 and reported that the incidence of PTB in Hefei city decreased from 57.96 per 100,000 population in 2009 to 31.04 per 100,000 population in 2020 with the high-high incidence cluster gradually decreasing in rural areas. Through targeted prevention and control strategies of PTB conducted in low-epidemic areas, the incidence of PTB had been reduced from high to low incidence. The epidemiological characteristics and prevention and control strategies of PTB in low-epidemic areas can provide a reference for the formulation in high-epidemic areas in the future to accelerate the end of PTB epidemic.

This study aimed to investigate the temporal trends and spatial patterns of PTB incidence in a low-epidemic are of China, Wuxi city in Jiangsu Province, from 2005 to 2020 through epidemic characteristics analysis and spatial analysis at the subdistrict level.

## Methods

### Study Area

**Figure 1:**
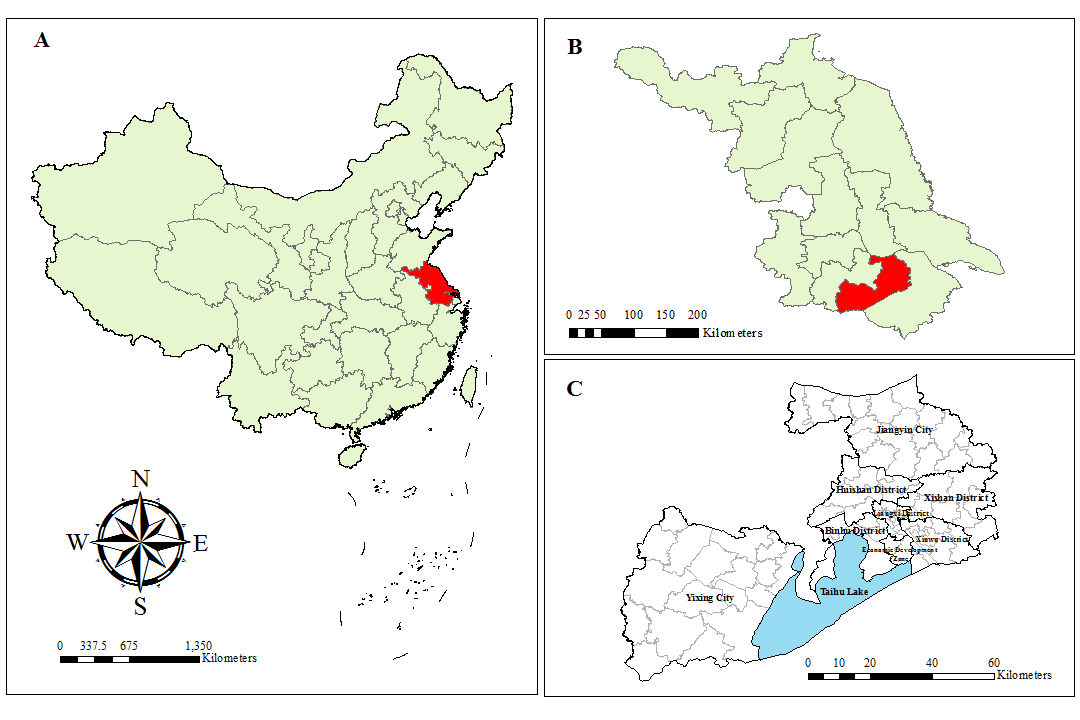
Location of the study area. The location of (A) Jiangsu Province in China, (B) Wuxi city in Jiangsu Province, and (C) 6 districts and 2 county-level cities in Wuxi city.

Wuxi city, with monsoon and maritime climate, is located in southern Jiangsu Province on the eastern coast of China (Figure 1). It covers an area of 4627.5 km^2^ and is composed of 6 districts and 2 county-level cities (Jiangyin city and Yixing city) with a total population of 7.5 million persons. It has a highly developed economy and emerged among the top-ranking cities in China in 2020 with a per capita gross domestic product exceeding US $20,000 [19].

### Data Collection

The Tuberculosis Information Management System is one of the components of the Notifiable Infectious Disease Reporting Information System. Since 2004, the system was implemented in all cities of China, and every PTB case has been required to report to this system in real time. In this study, the data of PTB cases from 2005 to 2020 in Wuxi city were obtained from the Tuberculosis Information Management System. Information about PTB cases included age, gender, address, as well as medical information such as drug resistance, classification (primary treatment or retreatment), and outcome. Demographic data were collected from the Statistical Yearbook of Wuxi.

### Statistical Analysis

Descriptive statistics were used to illustrate the characteristics of the population distribution. R software (version 4.0.3; The R Foundation) was used to investigate the level of statistical significance.

### Temporal Analysis

The joinpoint regression model [20] was used to identify the changes in the secular temporal trend of PTB in Wuxi city from 2005 to 2020. In the model, log-transformed PTB incidence rates and notification years were identified as the response variable and independent variable, respectively. The annual percent change, the average annual percent change, and the corresponding 95% CIs were obtained through joinpoint regression analysis. The best-fitted model was identified using the Bayesian Information Criterion (BIC). The equation for computing the BIC for a k-joinpoint model is as follows:


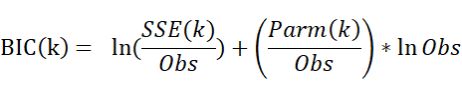


Where *SSE* is the sum of squared errors of the k-joinpoint regression model, *Parm*(*k*)=2*(k+1) is the number of parameters of the k-joinpoint model, and *Obs* is the number of observations. The k-joinpoint model with the minimum value of BIC(k) is selected as the final model. The joinpoint regression model was developed by the Joinpoint Regression Program (version 4.9.0.0; March 2021) from the Surveillance Research Program of the US National Cancer Institute.

### Spatial Analysis

Kernel density analysis [21] was used to explore the spatial distribution characteristics of PTB cases. The basic idea of Kernel density analysis is that geographical events have a high probability of occurrence in areas with high spatial point density and low probability in areas with low spatial point density, which can be used to explore hot spots of diseases. The density value was calculated on the basis of the following formulas:


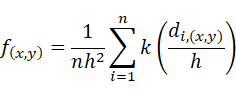


Where *f*_(_*_x_*_,_*_y_*_)_ represents the density value of the spatial coordinate position (x,y), *n* represents the number of PTB cases within the distance scale, *h* represents the bandwidth, *k* is the density function, *d_i_*_(_*_x_*_,_*_y_*_)_ represents the distance from a PTB case *i* to (x,y). ArcGIS software (version 10.4; ESRI) was used for Kernel density analysis of the PTB spatial distribution characteristics in Wuxi city.

### Cluster Analysis

Hot spot analysis (Getis-Ord Gi*) [22] was used to identify the clusters of PTB incidence rate in Wuxi city. First, the addresses were aggregated to the subdistricts, and the incidence rate of PTB was calculated. Then, the Getis-Ord Gi* statistic was analyzed for PTB incidence of subdistricts at different times. The Getis-Ord local statistic is given as follows:


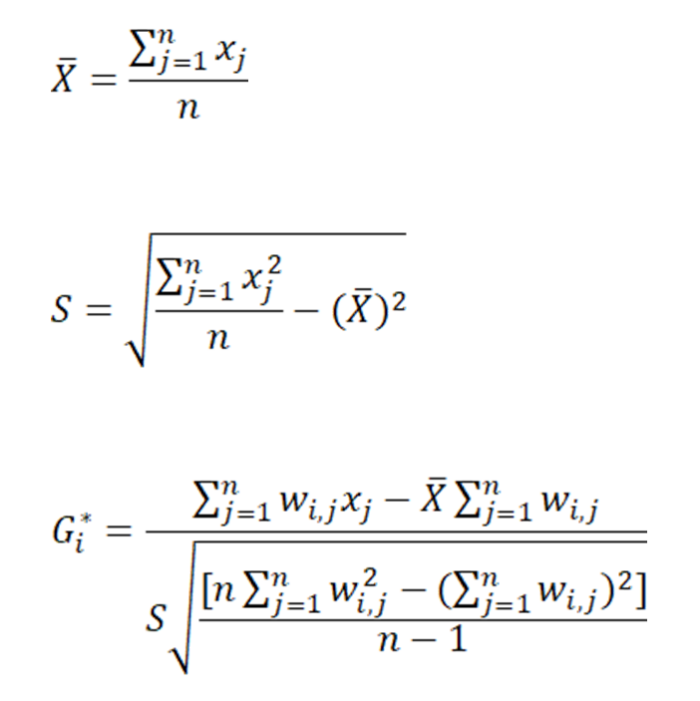


where *x_j_* is the attribute value for feature *j*, *w_i_*_,_*_j_* is the spatial weight between feature *i* and *j*, *n* is equal to the total number of features.

The resultant *z* scores and *P* values showed that high or low PTB incidence rates cluster spatially. A high *z* score with a small *P* value for the incidence indicated a spatial clustering of high incidence rates. A low negative *z* score with a small *P* value indicated a spatial clustering of low incidence rates. A *z* score near 0 indicated no apparent spatial clustering of PTB incidence rates. The Getis-Ord Gi* statistic was also analyzed using ArcGIS software (version 10.4; ESRI), and 999 permutations were conducted to ensure the statistical significance at 95% CIs.

## Results

### Demographic Characteristics

A total of 37,592 cases of PTB were registered during 2005 and 2020 in Wuxi city, with an average annual incidence rate of 34.6 per 100,000 population. Among the reported PTB cases, pathogen-positive PTB accounted for 50.5% (18,987/37,592) of cases, and the incidence was 17.5 per 100,000 population. As shown in Table 1, the incidence in men was more than twice that in women (*χ*^2^_1_= 6414.4, *P*<.001), with rates of 48.6 and 20.0 per 100,000 population, respectively. Although in the past 15 years, the largest number of notified PTB cases occurred in the age group of 20-39 years (14,754/37,592, 39.2%), the population older than 60 years had the highest incidence rate of 59.0 per 100,000 population, which was 1.4 times that of the 20-39–year age group.

**Table 1:** The number and incidence of pulmonary tuberculosis with different demographic characteristics in Wuxi city, 2005-2020.

Characteristics		Males		Females		Total	
		Cases, n	Incidence^a^	Cases, n	Incidence	Cases, n	Incidence
**Age group (years)**							
	0-19	1213	11.4	73	0.8	1946	9.6
	20-39	9787	54.0	4967	29.4	14,754	42.1
	40-59	7974	43.5	2578	14.8	10,552	29.5
	≥60	8061	94.1	2279	25.4	10,340	59.0
**Pathogenic classification**							
	Positive	13,865	24.9	5122	9.7	18,987	17.5
	Negative	12,665	22.8	5169	9.8	17,834	16.4
	Unknown	505	0.9	266	0.5	771	0.7
	Total	27,035	48.6	10,557	20.0	37,592	34.6

^a^Average annual incidence rate per 100,000 population.

### Temporal Distribution

**Figure 2:**
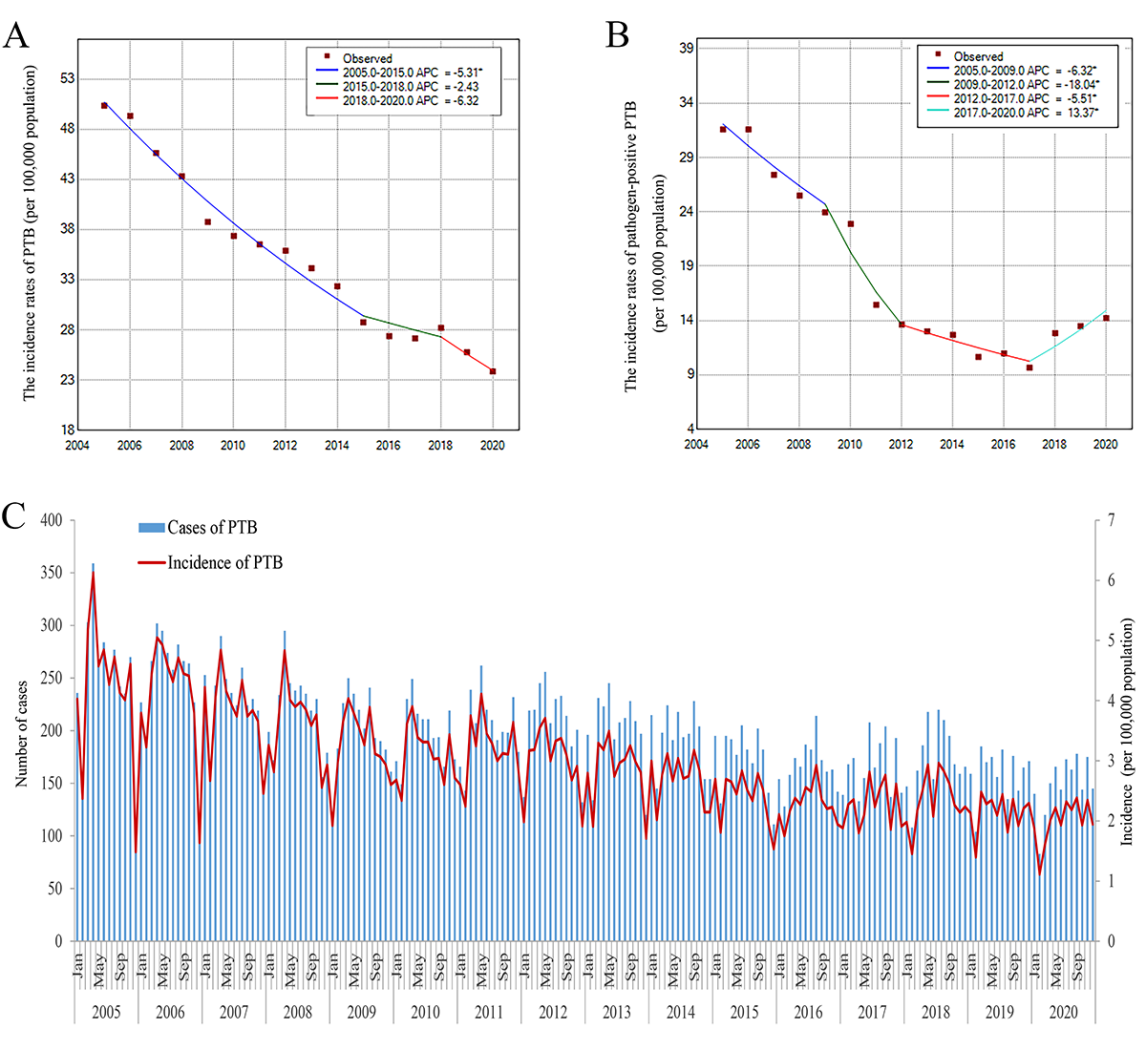
Temporal trend of pulmonary tuberculosis (PTB) and pathogen-positive PTB in Wuxi city, 2005-2020.

As shown in Figure 2A, the incidence rate of PTB in Wuxi city decreased from 50.4 per 100,000 population in 2005 to 23.9 per 100,000 population in 2020, with an average annual percent change of –4.9% (95% CI –6.8% to –2.9%). PTB incidence, with the implementation of the DOTS strategy and the Floating Population TB Control Pilot Project of the Global Fund TB Control Program in Wuxi, drastically declined during 2005 and 2015 with an annual percent change of –5.3% (95% CI –6.0% to –4.6%). Subsequently, PTB incidence decreased slowly by –2.4% every year. However, since 2018, the incidence in Wuxi has once again entered a period of rapid decline, with an annual percent change of –6.4%. The pathogenic positive PTB incidence showed different temporal patterns in Figure 2B. The pathogenic positive PTB incidence decreased from 31.6 per 100,000 population in 2005 to 9.7 per 100,000 population in 2017. However, there was an ascending trend in the incidence of pathogen-positive PTB from 2017 to 2020, with an annual percent change of 13.4% (95% CI 4.3%-23.2%). The seasonal trend of PTB incidence in Wuxi city gradually changed from a single peak to a bimodal pattern, resulting in no obvious incidence peak. But, a trough of PTB incidence rates was observed during the entire study period (Figure 2C).

### Spatial Distribution

**Figure 3:**
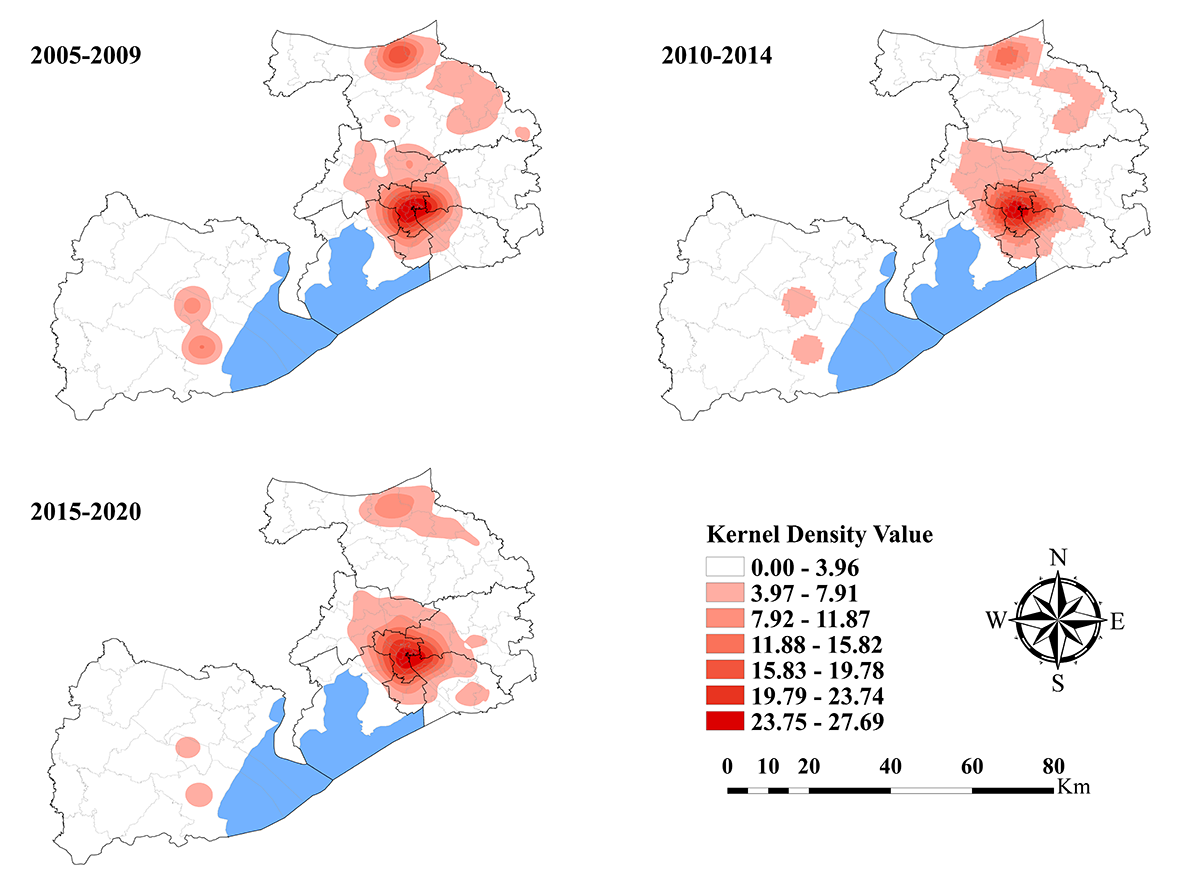
The spatial distribution of pulmonary tuberculosis cases in Wuxi city at the county level, 2005-2020.

The spatial distribution of PTB cases in Wuxi city at the county level during 2005-2020 is shown in Figure 3. The cases were mainly concentrated in the city center, namely Liangxi District and the surrounding subdistricts or towns. The same spatial characteristic also existed in 2 county-level cities, Jiangyin city and Yixing city, where PTB cases were concentrated in downtown areas. On comparing the Kernel density of cases in 2005-2009, 2010-2014, and 2015-2020, the high-PTB-density areas gradually shrank, and the Kernel density value also decreased.

### Cluster Analysis

**Figure 4:**
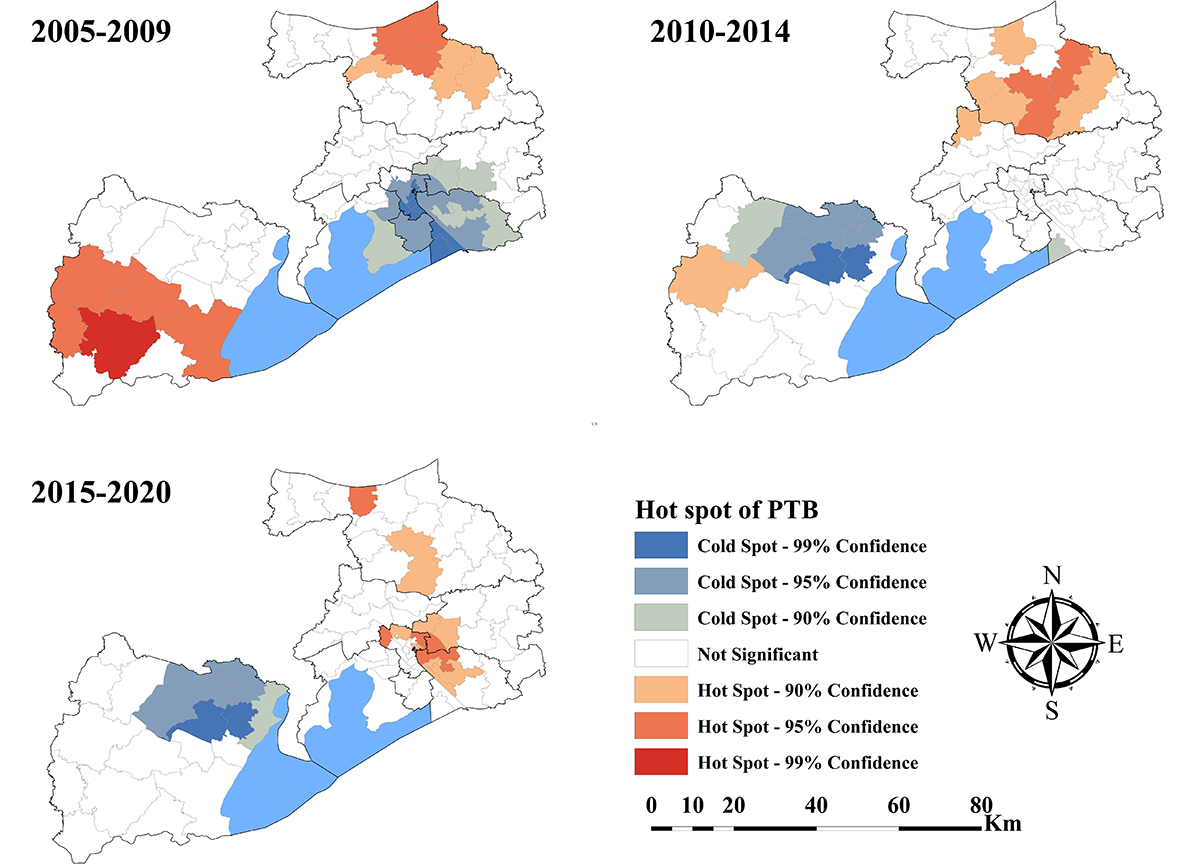
The spatial cluster of the pulmonary tuberculosis (PTB) incidence rate in Wuxi city at county level, 2005-2020.

Figure 4 shows the results of PTB incidence cluster analysis of the Local Getis Gi* statistic. In 2005-2009, we found that the significant hot spots were in the northeast of Jiangyin city and southwest of Yixing city. Significant cold spots were located in Liangxi District, Economic Development Zone, and the surrounding subdistricts or towns. According to our results in 2010-2014, the significant hot spots were mainly located in the eastern and southern regions of Jiangyin city. However, the significant cold spots were mostly in northern Yixing city. During 2015-2020, the location of the PTB incidence cold spots had not changed much and was still in Yixing city. However, PTB incidence hot spots were mainly detected in the Wuxi city center and 2 subdistricts of Jiangyin city.

## Discussion

### Principal Findings

During the 16-year period of 2005 to 2020, the PTB notification rates of Wuxi city decreased from 50.4 to 23.9 per 100,000 population at an average rate of –4.9% per year. The decline was higher than that in China (–3.8%) during the same period [8]. In addition to the implementation of national measures such as the DOTS strategy, national TB prevention strategies, control plans, and government commitments, 3 factors could have contributed to the difference. First, the lower latent infection rate of TB indicates a lower TB incidence. According to the results of a population-based, multicenter, prospective cohort study by Gao et al [23], the prevalence of latent TB infection in the eastern China plains was 13.5%, which was lower than that in the central and western regions of China. Further, the well-developed medical conditions and convenient access to medical care in the eastern plain regions have further halted PTB transmission [19]. The second factor was the implementation of the Floating Population Tuberculosis Control Project from 2007 to 2011, which was funded by the Fifth Round-China Global Fund TB program. The effects of the program were consistent with studies conducted in other cities [24,25]. By providing free inspection and grants, supervising the entire treatment process, the cure rate among patients treated for PTB in the floating population increased, and the transmission of PTB in the population reduced. The last factor was introducing and promoting the use of new technology in the diagnosis of TB. From 2018 to 2019, loop-mediated isothermal amplification was used free of charge to diagnose PTB in all TB-designated (specialized) hospitals in Wuxi city, mainly for further testing of sputum smear–negative specimens. However, the TB-designated (specialized) hospitals in Wuxi provide free Xpert MTB/RIF molecular tests for all individuals suspected with TB since 2020. With the application of molecular techniques, more pathogen-positive PTB cases were promptly detected and treated. It was essential for reducing the contagiousness of pathogen-positive PTB cases and controlling PTB transmission. Thus, the incidence of pathogenic positive PTB increased from 9.7 per 100,000 population in 2017 to 14.2 per 100,000 population in 2020, with an annual percent change of 13.4%; nonetheless, the overall incidence of PTB decreased at a rate of 6.3% per year in Wuxi city.

During the study period, the seasonal pattern was gradually unobvious—this may be explained by the lower incidence rate of PTB in Wuxi city. However, similar to other studies in China [9,22,26], our results show an incidence trough in February, especially in 2020. The reason for the decreasing rate is that Chinese people often prepare for the Lunar New Year in February, and citizens usually try to avoid seeking medical advice immediately. This is one of the reasons that an epidemic peak occurred in March and April. But in 2020, to control and prevent the outbreak of COVID-19, the Wuxi government also implemented a series of public interventions including community or home isolation, restriction of public traffic, health care staff reassignment [27-29], etc. It had seriously affected notification rates. In February 2020, the incidence rate of PTB in Wuxi city dropped by 35.3% compared to the average rate of the same period in 2017-2019.

According to our study, the population older than 60 years was the main target group of PTB with the highest incidence rate of 59.0 per 100,000 population, especially in males (94.1 per 100,000 populations). This finding was similar to those of other studies [14,16,22]. On the one hand, the older population harbors a high burden of the prevalence of latent TB infection, and on the other hand, the attenuation of immunity and underlying disease result in higher susceptibility to infection and reinfection in the older population [23,30,31]. With China's population aging rapidly, the prevention and control of TB in the older population will be a major challenge to eradicate TB.

Notably, spatial heterogeneity of PTB was observed in Wuxi city during 2005 and 2020. The results from the maps of Kernel density estimation analysis showed that PTB cases were mainly concentrated in the city center. This could be explained by the uneven distribution of the population in Wuxi city. With economic and social factors, the population is usually concentrated in the city center and the surrounding subdistricts or towns. Therefore, these densely populated areas were prone to the spread of PTB, which led to the accumulation of cases. According to PTB incidence cluster analysis, the hot spots of high-PTB-incidence areas gradually shifted from the suburbs to the city center of Wuxi city, which was similar to the spatial characteristics of PTB from 2009 to 2020 in Hefei city [18]. The results indicate that PTB control measures in the past decade have been very effective in rural areas. However, it is an urgent need for new PTB control strategies or projects to prevent PTB from spreading throughout the population in densely populated urban areas.

### Limitations

This study has several limitations. First, because of death or treatment in other areas, some cases were not registered in the official system. Second, the incidence rate of PTB in this study was based on the resident population, so the incidence may have been underestimated in some areas with a large floating population. Those people were not registered in Wuxi city owing to their return to their registered permanent residence to begin PTB treatment. Third, we only conducted analyses to explore the spatial and temporal characteristics of PTB. Further studies should be carried out to detect the risk factors of PTB in low-epidemic areas in China.

### Conclusions

This study identified temporal trends and spatial distribution of PTB incidence at the subdistrict level in a low-epidemic area of China, Wuxi city, from 2005 to 2020. The PTB incidence rate in Wuxi city has been declining rapidly with effective implementation of strategies and projects. The populated urban centers are expected to become key areas of PTB prevention and control, especially for the older population.

## Abbreviations

BIC: Bayesian Information Criterion
DOTS: directly observed treatment and short-course
PTB: pulmonary tuberculosis
TB: tuberculosis

## Data Availability

The data sets used and analyzed during the current study are available from the corresponding author on reasonable request.
